# Long-Term Olfactory Dysfunction in COVID-19 Patients: A Systematic Review

**DOI:** 10.7759/cureus.103143

**Published:** 2026-02-07

**Authors:** Artemis Zarkadi, Michail Katotomichelakis, Konstantinos Chaidas

**Affiliations:** 1 Otolaryngology - Head and Neck Surgery, School of Medicine, Democritus University of Thrace, Alexandroupolis, GRC; 2 Otolaryngology - Head and Neck Surgery, Imperial College Healthcare NHS Trust, London, GBR

**Keywords:** anosmia, coronavirus, covid-19, hyposmia, long covid, olfaction disorders, olfactory, parosmia, sars-cov-2, smell

## Abstract

Olfactory dysfunction (OD) emerged early in the COVID-19 pandemic as a prevalent and often persistent symptom. While most individuals recover within weeks, a significant proportion continue to suffer from long-term impairments, including both quantitative and qualitative sensory deficits. Our review aimed to summarize current evidence on long-term post-COVID-19 OD with a duration of at least three months, including prevalence, recovery trajectory, and prognostic factors. The PubMed and Scopus databases were searched for relevant studies up to August 2024 following Preferred Reporting Items for Systematic Reviews and Meta-Analyses (PRISMA) guidelines. Twenty-one studies were ultimately included, involving over 4,000 individuals. A remarkable proportion of patients continue to experience persistent dysfunction post-infection for a period ranging from several months to over two years. Qualitative disorders, such as parosmia and phantosmia, frequently appeared during recovery. Prognosis seemed to be related to age, initial severity, duration of OD, co-existing symptoms, and potentially sex. A consistent discrepancy between subjective reports and objective psychophysical test results was observed. Methodological heterogeneity limited comparability across studies. Olfactory dysfunction is a significant and often overlooked long-term complication of COVID-19. Standardized diagnostic criteria, validated outcome measures, and prospective longitudinal research are urgently needed to guide evidence-based management and improve patient outcomes.

## Introduction and background

Since the onset of the COVID-19 pandemic in December 2019, the severe acute respiratory syndrome coronavirus 2 (SARS-CoV-2) has caused an unprecedented global health crisis, infecting nearly 780 million people worldwide (WHO) and leading to significant morbidity and mortality [1]. Early clinical efforts focused primarily on managing the acute phase of COVID-19, aiming to alleviate immediate symptoms and reduce mortality rates. However, research has shown that many individuals experience persistent and sometimes debilitating symptoms long after the acute infection has resolved [2]. This condition, known as long COVID (or post-acute COVID-19 syndrome), is characterized by the continuation or development of new symptoms three months after the initial SARS-CoV-2 infection [3]. It encompasses a broad range of symptoms, including fatigue, respiratory difficulties, chemosensory disorders, cognitive impairment, and cardiovascular complications.

Among these diverse symptoms associated with long COVID, olfactory dysfunction (OD) has emerged as a significant concern. Disturbances in the sense of smell can manifest in the following two forms: quantitative deficits, such as anosmia (total loss of olfactory function without smell perception even if odors are very strong) and hyposmia (decreased olfactory function with smell perception if odors are strong), and qualitative distortions, including parosmia (distorted smell triggered by an odor source) and phantosmia (distorted smell occurring independently of an odor source). Although most individuals recover their sense of smell within a few weeks, a substantial subset suffers from long-term or permanent OD. Such persistent impairment can profoundly affect daily life, as the sense of smell is essential for emotional well-being, nutritional habits, and safety [4].

Despite the growing recognition of OD as a long-term consequence of COVID-19, the prevalence and persistence of these symptoms remain unclear [2]. Understanding the extent and nature of OD in the post-acute phase is crucial for effective patient management and care. This systematic review aimed to provide a comprehensive overview of the current literature on long-term olfactory dysfunction in adults, focusing on cases where symptoms persist beyond three months following SARS-CoV-2 infection. Our study focused on data regarding OD prevalence and recovery rates for both quantitative and qualitative changes, as well as associated prognostic factors.

## Review

Materials and methods

This systematic literature review was conducted in accordance with the Preferred Reporting Items for Systematic Reviews and Meta-Analyses (PRISMA) 2020 guidelines. The PubMed and Scopus databases were systematically searched for relevant articles published through August 2024. The search was conducted using the following keywords: "COVID-19" OR "coronavirus" OR "SARS-CoV-2" AND "anosmia" OR "olfactory dysfunction" OR "loss of smell" AND "recovery" AND "long COVID." To be eligible for inclusion in this review, studies had to provide data regarding olfactory dysfunction in adults aged 18 years and older with a confirmed previous diagnosis of COVID-19 based on polymerase chain reaction (PCR) testing. References of all full-text articles were manually searched, and additional relevant articles were also included. Participants were assessed utilizing subjective methods, psychophysical tests, or a combination of both, with a minimum follow-up period of three months. The exclusion criteria included non-English articles, reviews, editorial letters, and conference abstracts. Additionally, all studies involving non-human participants were excluded. To ensure consistency, two authors (AZ and KC) independently performed the article search, article selection, and data extraction, using standardized data forms, with a third author (MK) verifying the collected data. Disagreements were addressed and resolved by consensus. The quality of each study was evaluated by using the Grading of Recommendations Assessment, Development and Evaluation (GRADE) system [5].

Results

Search Results and Article Selection

**Figure 1:**
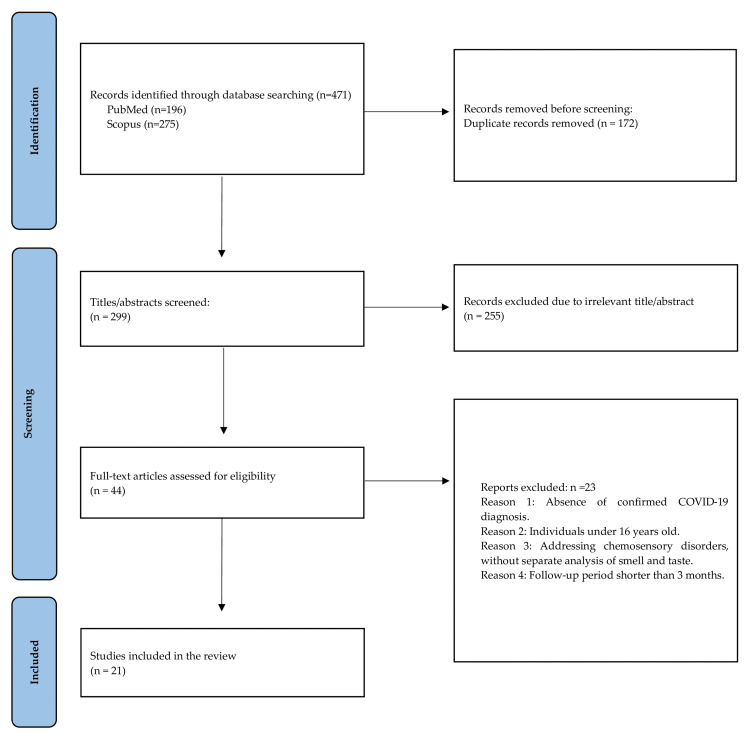
PRISMA flowchart of articles search and selection. PRISMA: Preferred Reporting Items for Systematic Reviews and Meta-Analyses, n: number of studies

The article selection and eligibility process is shown in Figure 1. At the initial electronic database search, a total of 471 articles were identified. After duplicate record removal, the titles and abstracts of 299 studies were screened to assess their applicability. Subsequently, full-text articles of 44 potentially eligible studies were evaluated against the predefined inclusion criteria. Reasons for exclusion were as follows: (1) absence of a confirmed COVID-19 diagnosis, (2) study participants under 18 years old, (3) data presentation of chemosensory disorders without separate analysis of smell and taste, and (4) follow-up period shorter than three months. Finally, 21 studies were included in this review.

Included Study Characteristics

Table 1 shows the characteristics of all included studies, outlining variations with regard to study type, assessment methods, follow-up periods, and main findings. Among the studies, 17 were prospective, one was retrospective, one employed a mixed-methods design combining both prospective and retrospective elements, and two were cross-sectional. Ten studies used objective psychophysical assessments, while 11 relied on subjective self-report methods.

**Table 1:** Individual study characteristics. SD: standard deviation; m: male; f: female; GRADE: Grading of Recommendations Assessment, Development and Evaluation; VAS: visual analog scale; OD: olfactory dysfunction; SNOT-22: Sino-Nasal Outcome Test 22; UPSIT: University of Pennsylvania Smell Identification Test; B-SIT: Brief Smell Identification Test; ISARIC: International Severe Acute Respiratory and Emerging Infection Consortium; B-SITC: Brief Smell Identification Test for Chinese; n: number; HRS: Hyposmia Rating Scale

Studies	Country	Study type	Assessment methods	Patients, n	Age (years) mean±SD/median (range)	Sex, m/f	Follow-up	Main outcomes	Study quality (GRADE)
Boldes et al. (2024) [6]	Israel	Prospective observational study	Online questionnaire, VAS	40	51±12.6	19/21	2, 9, 24 months	At 24 months: 92% had some improvement in olfactory function, but 47% had full recovery, 53% had persistent OD.	Very low
Boscolo-Rizzo et al. (2023) [7]	Italy	Prospective observational study	SNOT-22	253	48 (38-56)	95/158	12, 24 months	At 24 months: 33/253 patients (13%) had smell complaints, 20/253 patients (7.9%) had quantitative OD (13 patients had parosmia, 12 patients had phantosmia).	Very low
Callejón-Leblic et al. (2022) [8]	Spain	Prospective cohort study	Questionnaire, VAS, UPSIT	102	46.8±13.9 (23-89)	32/70	12 months	According to VAS: 58/102 patients (56.9%) had normosmia, 27/102 patients (26.5%) had mild loss of smell, 8/102 patients (7.8%) had moderate loss of smell, and 8/102 patients (7.8%) had severe loss of smell. 28 patients (27.5%) had parosmia. According to UPSIT: 12/69 patients (17.4%) had normosmia, 30/69 patients (43.5%) had mild, 15/69 patients (21.7%) had moderate, 7/69 patients (10.1%) had severe microsmia, 5/69 patients (7.2%) had anosmia.	Very low
Ciofalo et al. (2022) [9]	Italy	Prospective observational study	Telephone interview, VAS, B-SIT	162	57.0 (48.8-63.0)	82/80	7, 14, 21, 28, 90, 180 days	At 6 months: according to VAS, 44/162 patients (27.2%) had anosmia; among those 44 patients, according to B-SIT, 44.2% had anosmia and 55.8% had hyposmia.	Very low
Fernandez et al. (2022) [10]	Italy	Retrospective observational study	Modified COVID-19 anosmia reporting tool for clinicians via e-mail	146	40.2±12.1	51/95	Mean: 5.6±2 months	29/146 patients (19.9%) had partial recovery, 23/146 patients (15.8%) had no recovery.	Very low
Ferreli et al. (2022) [11]	Italy	Prospective observational cohort study	Telephone survey	132 (99 reported OD)	Mean: 49.2	58/73	1, 3, 6, 9, 12, 18 months	At 3 months: 80/99 (80.8%) patients had full recovery. At 18-months: 86/99 patients (86.9%) had full recovery, 13 patients (13.1%) had persistent OD. Among the 13 patients, 3/13 had parosmia (23.1%).	Very low
Kalak et al. (2022) [12]	Israel	Prospective cohort study	Open-ended questionnaire	166	52.1±16.8 (19-86)	83/83	3, 18 months	At disease onset: 17.5% had anosmia. At 3 months: 5.4% had anosmia. At 18 months: 0% had anosmia.	Low
Lamb et al. (2023) [13]	USA	Prospective cross-sectional study	UPSIT	86	Mean: 50.5 (18-89)	26/60	3 till >24 months	At 12 months: 44.8% had OD. At >24 months: ~20% had OD.	Low
Liu et al. (2024) [14]	Singapore	Both retrospective and prospective arms	Phone interview, self-administered B-SIT	40	Median: 40	29/11	36 weeks	According to the telephone interview, 76% with full recovery. According to BSIT, 51.5% had full recovery. Four patients had parosmia and 3 patients had phantosmia.	Very low
McWilliams et al. (2022) [15]	USA	Prospective survey	Questionnaires via email	946	43.8±13.9 (18-82)	178/753	14 days, 1, 3, 6, 24 months	At ≥3 months: 38.7% had full recovery, 51% had partial recovery, 10.3% had no recovery. At ≥1 year: 38.9% had full recovery, 51.4% had partial recovery, 9.6% had no recovery. At ≥2 years: 38.2% had full recovery, 54.3% had partial recovery, 7.5% had no recovery. Among 579 patients who did not report complete recovery, 64.9% reported smell distortion, 38.9% reported smelling strong odors only, 32.6% reported phantosmia.	Very low
Otte et al. (2021) [16]	Germany	Prospective study	Sniffin' Sticks identification test	26	45±2.06	15/11	200 days (±2.52)	19/26 patients (73.1%) had normosmia, 7/26 patients (26.9%) had hyposmia.	Very low
Perez et al. (2024) [17]	Spain	Cross-sectional study	Phone interview, ISARIC COVID-19 follow-up survey	694	47 (33-60)	294/400	4.7-24 months	115/694 patients (16.6%) had partial recovery. Of them, 47/115 patients (40.9%) had anosmia.	Very low
Petrocelli et al. (2021) [18]	Italy	Prospective study	Phone interview, ethyl alcohol olfactory threshold, and discriminative function	300	43.6±12.2 (33-53)	75/225	1, 2, 3, 6 months	At baseline: 190/300 patients (63.3%) had OD. At 6 months: 81/300 patients (27%) had persistent OD, including anosmia in 15/300 patients (5%).	Low
Prem et al. (2022) [19]	Austria	Prospective study	Sniffin' Sticks identification test, 27-item Candy Smell Test, questionnaires	102	Mean: 38.8 (18-68)	31/71	Mean: 216 days (111-457)	23.5% had normosmia, 76.5% had OD (4% had anosmia, 72.5% had hyposmia). At follow-up: 73.5% had improvement, 5.9% had deterioration, 20.6% had no change in OD.	Very low
Rass et al. (2022) [20]	Austria	Prospective, multicenter, longitudinal cohort study	Interview, Sniffin' Sticks identification test	81	54 (47-64)	48/33	3, 12 months	At 12 months: 12/81 patients (15%) had self-reported OD, 41/81 (51%) had objective OD.	Very low
Riestra-Ayora et al. (2021) [21]	Spain	Prospective case-control study	VAS	320	Control-group mean: 46.5 (20-64). Case-group mean: 41.62 (18-65)	58/262	6 months	Case-group at baseline: 125/195 patients (64.1%) had OD. Case group at 6 months: 73/195 patients (37.4%) had complete recovery, 38/195 patients (19.5%) had partial recovery, and 14/195 patients (7.2%) had no recovery. 24% of patients had OD reported dysosmia - most commonly parosmia (73%).	Moderate
Larijani et al. (2022) [22]	Iran	Prospective cohort study	Face-to-face or phone interview	254	41 (35-49)	137/117	12-24 weeks, >24 weeks	At 12-24 weeks: 10/82 patients (12.2%) had anosmia. Beyond 24 weeks: 7/54 patients (13%) had anosmia.	Very low
Schambeck et al. (2021) [23]	Germany	Prospective observational cohort study	Sniffin' Sticks identification test, questionnaire	44	41 (23-62)	15/29	100, 244 days	At 100 days: 8/44 patients (18.2%) had OD (6/44 patients had parosmia, 4/44 patients had phantosmia, 3/44 patients had hyposmia). At 244 days: 12/44 patients (27.3%) had OD (6/44 patients had parosmia, 5/44 patients had phantosmia, 4/44 patients had hyposmia)	Very low
Turk et al. (2022) [24]	Turkey	Cross-sectional survey	Questionnaire, VAS	77	42±14.6	36/41	12-14 months	12/77 patients (15.6%) had loss of smell, 3/77 patients (3.9%) had cacosmia, and 5/77 patients (6.5%) had parosmia.	Very low
Wang et al. (2022) [25]	China	Prospective study	Telephone survey	11	34 (23-46)	9/2	1, 6, 12 months	At 6 months: 6/11 patients (54.5%) had full recovery, 3/11 patients (27.3%) had partial, 2/11 patients (18.2%) had no improvement. At 12 months: 9/11 patients (81.8%) had full recovery and 2/11 patients (18.2%) had partial recovery.	Very low
Zhu et al. (2021) [26]	China	Prospective study	B-SITC, HRS	95	49.22±14.74	49/46	16, 21 weeks	At 16 weeks, according to B-SITC, 22/95 patients (23.2%) had hyposmia. According to HRS, 26/82 patients (31.7%) had hyposmia. Hyposmia rates decreased from 16 weeks (34.1%) to 21 weeks (24.4%) among the 41 patients who completed both visits.	Very low

Variation in Study Methodology

This review demonstrates a wide variety of methodological approaches used to evaluate COVID-19-related OD across studies. The studies include both retrospective data, such as medical record reviews, and prospective follow-ups of patient cohorts for varying periods, ranging from over three months to over two years. Some studies adopted a mixed design, combining retrospective data collection with prospective follow-up. Additionally, there are cross-sectional studies analyzing data collected at specific time points, as well as case-control studies. Sample size and patient characteristics, including age, sex, and disease severity, also varied across studies, which could account for some of the variability in reported rates.

Both subjective and objective methods were used for the evaluation of olfactory function, with a wide variation between the studies. Subjective assessments were primarily based on questionnaires, telephone interviews, and the visual analog scale (VAS) for quantifying symptom severity. Specialized symptom reporting tools such as the SNOT-22 and the ISARIC survey were also utilized [7,17]. Objective assessments included the application of psychophysical tests for smell. Specific olfactory tests used include the Burghart Sniffin' Sticks (including identification, discrimination, and threshold tests) [16,19,20,23], the University of Pennsylvania Smell Identification Test (UPSIT) [8,13], the 27-item Candy Smell Test (27-CST) for retronasal olfactory function assessment [19], and the Brief Smell Identification Test (B-SIT) [9,14,26]. Overall, the heterogeneity in assessment methods and data analysis makes it challenging to compare findings across studies and underscores the need for consensus on terminology and study design.

Prevalence and Recovery

Sudden smell impairment is a frequent symptom during the acute phase of COVID-19, with a great variability in reported rates between the published reports and a prevalence of up to 85.2% [9]. The trajectory of olfactory recuperation also exhibits remarkable variability. Most patients report symptom resolution within the first few weeks or months [9,14,25,26]. Ciofalo et al. observed the onset of smell recovery commencing at 14 days [9]. Similarly, another study indicated that 76% of patients with OD recovered within a five-week period [14].

However, a significant proportion of patients experience persistent dysfunction for a period longer than three months, which can be extended to over two years [7,15,17]. Riestra-Ayora et al. observed that 11% and 30% of participants displayed no recovery and partial recovery of OD, respectively, at six months [21]. Another study revealed that 44.8% of patients still exhibited OD one year post-infection and approximately 20% beyond two years [13]. Likewise, Ferreli et al. revealed that 13.2% of patients continued to experience OD at 18 months [11]. Interestingly, studies evaluating patients two years post-infection showed persistent olfactory impairment in more than half of the patients [6,15]. McWilliams et al. demonstrated that out of 264 individuals with at least two-year follow-up, 54.3% experienced only partial recovery, and 7.5% showed no recovery [15]. Some patients achieved full recovery after one year, underscoring the potential for delayed recuperation. On the other hand, a study by Boscolo-Rizzo et al. identified no cases of complete anosmia at two years, although 13% of patients continued to experience partial loss of smell [7].

Qualitative Disorders

Beyond quantitative impairment, qualitative changes in olfaction are frequently reported and may persist even subsequent to initial recovery. Parosmia is the most common symptom reported by patients experiencing qualitative OD and can be enduring for even more than two years [6,8,11]. In addition to parosmia, the presence of cacosmia (unpleasant odors) was also documented in a subset of patients with at least one year of follow-up [24]. According to a study, among individuals with incomplete olfactory recovery, 64.9% reported smell distortions and 32.6% experienced phantosmia [15]. Callejón-Leblic et al. suggested that parosmia is often associated with severe initial olfactory impairment and moderate to severe baseline gustatory dysfunction [8]. Notably, phantosmia and parosmia may arise months after the initial resolution of symptoms or even after a period of perceived complete recovery, indicating a complex and sometimes delayed onset of these distortions [23]. Additionally, the severity of a qualitative disorder such as parosmia can worsen over time [19].

Assessment Methods

A consistent and significant finding across studies is the substantial discrepancy between self-reported (subjective) olfactory function and objectively measured results. This indicates that self-assessment often underestimates the true extent of persistent OD. Liu et al. demonstrated that although 78.8% of participants reported normal olfaction, only 51.5% achieved normal scores on objective testing with B-SIT, revealing a low correlation between the two measures [14]. Similarly, another study demonstrated that among patients who self-reported a normal sense of smell, 75% still exhibited some degree of alteration on UPSIT, with the overall correlation between UPSIT and self-reported VAS scores being only moderate [8]. Rass et al. further emphasized this disparity, revealing a marked difference between self-reported hyposmia (15%) and objective hyposmia (51%), one year following SARS-CoV-2 infection [20]. A study employing Sniffin' Sticks for objective assessment found that while 73.5% of patients reported subjective improvement at follow-up, 72.5% and 4% remained objectively hyposmic and anosmic, respectively [19]. Overall, multiple studies consistently demonstrate a higher prevalence of OD when psychophysical tests are utilized compared to self-reports. These findings emphasize the indispensable role of objective psychophysical testing in accurately diagnosing and monitoring post-COVID-19 OD, as subjective assessments may not fully capture the lingering impairment.

Prognostic Factors

Various factors have been examined regarding their influence on OD recovery. Age consistently emerges as a significant factor, with reports of notably higher rates of complete recovery among individuals under 40 years old than among those over 40 years [8,15]. Increasing age was identified as an independent risk factor associated with objective long-term OD [7,8,18]. In addition to older age, the presence of parosmia at baseline was also linked with a negative impact on olfactory recovery [7]. The association between sex and olfactory function recovery presents mixed findings; Perez et al. observed that males were more likely to experience complete recovery than females [17], whereas another study found no correlation between sex and recovery [11]. The initial severity and duration of OD are crucial considerations; Ferreli et al. demonstrated that delayed recovery was correlated with severe OD at presentation [11], whereas the duration of anosmia was identified as the sole significant factor associated with incomplete or absent recovery [10]. A study by Callejón-Leblic et al. additionally specified that smell loss duration exceeding four weeks diminished the likelihood of recovery [8].

Moreover, concomitant symptoms may influence outcomes. A greater prevalence of nasal obstruction and headache was noted among patients with anosmia [9]. Another study identified nasal obstruction and dyspnea as risk factors for persistent symptoms; however, having experienced fever at baseline might mitigate the risk of ongoing chemosensory dysfunction [8]. On the other hand, the severity of initial COVID-19 illness and the responsible dominant variant appear to exhibit minimal influence on OD and its recovery [8,13].

Discussion

Olfactory dysfunction is a highly prevalent symptom during the acute phase of COVID-19 illness, presenting in over 50% of patients [27]. Interestingly, OD was found to be one of the most discriminating patient-reported symptoms of long COVID in the RECOVER-Adult cohort of 9,764 participants [28]. Although initially regarded as a minor symptom, persistent OD has a significant impact on patients' quality of life, impacting not only sensory perception but also daily activities and overall health [29]. Smell is vital for safety purposes, food enjoyment, and even mental health, as well as career stability for individuals engaged in professions that rely heavily on olfactory senses, such as chefs or firefighters [4]. Therefore, understanding the factors that influence its long-term prevalence, duration, and severity is essential for optimizing patient management.

A prominent theme emerging from the literature is the variability of recovery patterns and the considerable proportion of patients experiencing long-term or incomplete resolution of symptoms [2]. Our review indicates that while the majority of COVID-19 patients recover their sense of smell relatively quickly, a significant proportion experience persistent or incomplete recovery, with initial severity and certain demographic and clinical factors associated with the recovery course. The use of objective tests is crucial for accurate diagnosis and monitoring, as subjective reports may underestimate the dysfunction. Qualitative alterations also represent a significant issue that can manifest even at a later stage.

The overall prevalence of COVID-19-related OD was calculated by previous reviews. Specifically, three studies by Wu et al., Tong et al., and Ahmad et al. reported a prevalence of 53.56%, 52.73%, and 59.69%, respectively, collectively emphasizing the high frequency of OD among individuals who have contracted COVID-19 [27,30,31]. This is further supported by a comprehensive meta-analysis of over 27,000 patients, which reported a pooled prevalence of 47.85% [32]. However, the above studies evaluated the presence of OD shortly after the initial infection without focusing on the long-term persistence. On the other hand, a literature review by Dias et al. revealed inconsistency in delayed (>6 months) recovery rates. Based on self-assessment, only about 5% of the patients experienced OD for longer than six months post-infection, but this percentage appeared to be much higher following psychophysical testing [33].

This study is the first systematic review focusing on COVID-19-related olfactory function alone and assessing only studies with long-term follow-up of three or more months. It demonstrates that long-term OD is not uncommon, with most studies reporting a prevalence of at least 27% at six months after infection [9,10,18,21,22,25], whereas it also remains high even after one or two years [6-8,13,15,20,24,25]. Studies with repeated olfactory assessments of the same patient cohort over a long-term period reveal that a remarkable proportion of patients experience a late recovery ranging from six months to two years [6,7,11-13,15,20,22,25].

Despite late OD recovery, there is a significant number of patients experiencing persistent OD after two years. The reported rates varied across studies, ranging between 13.3% and 61.8% [6,7,13,15]. An interesting review and meta-analysis by Rahmati et al. examined the prevalence of persistent symptoms three years following initial SARS‐CoV‐2 infection [2]. Although they evaluated OD among various symptoms, their results strongly correspond with our findings. Specifically, they indicated that olfactory disorders persist in 7% of patients at three years post-infection.

A large variability in reported rates of recovery is noted among studies, which possibly reflects their heterogeneity. The differences refer to study design, sample size, parameters used, outcome measures, and duration of follow-up. Patient characteristics, including age, sex, and disease severity, also varied across studies. Moreover, most studies were single-center and were conducted in different countries, which may have also played a role in the findings. For instance, a significantly higher prevalence of OD induced by the Omicron variant was reported in European populations (11.7%), compared to a range between 1.9% and 4.9% reported in all other populations [34].

In addition, a key similarity between our study and existing literature is the consistent discrepancy between subjective patient reports and objective clinical assessment of OD. Objective smell tests appear to detect OD in a higher percentage of patients than self-reports. Saniasiaya et al. observed a markedly higher prevalence of OD when employing objective testing methods, specifically, 72.10% compared to 44.53% based on subjective reports [32]. This may be due to patients' lack of awareness regarding the extent of the impairment or their adaptation to the altered sensation. Some investigators report a degree of correlation between subjective and objective test scores, but their association was described as weak to moderate [8]. Overall, objective testing is considered indispensable for accurate evaluation in order to prevent underestimation of the true prevalence of persistent OD, particularly in long-term follow-up. However, despite the above variations, it is remarkable that all studies with longer follow-up consistently demonstrate the presence of ongoing recovery for years after COVID-19 infection.

Several factors have been linked with persistent OD post-COVID-19 infection. Relevant studies included in our review indicate that older age is strongly associated with lower rates of recovery, and increasing age appears to be an independent factor associated with prolonged smell impairment, consistent with findings from other studies [2,33]. This could be attributed to age-related gradual deterioration of the immune system or pre-existing comorbidities [35]. Initial severity and duration of OD post-infection were also identified to influence OD recovery. On the other hand, the role of other parameters is not clear. Female sex has been associated with a worse recovery course of OD [2,15,33], which could be due to hormonal and immunological differences, although another study showed no such correlation [11]. Furthermore, research showed mixed findings regarding other potential prognostic factors, such as the severity of initial SARS-CoV-2 infection and the type of variant responsible. Interestingly, a review and meta-analysis by von Bartheld and Wang showed that the Omicron variant was associated with a substantially lower prevalence of OD, with a two- to 10-fold reduction compared to earlier variants such as alpha and delta [34].

In order to identify the reasons for prolonged COVID-19-related OD, it is important to explore the underlying pathophysiology. The suggested pathophysiological mechanisms in those patients are multifactorial. Typically, upper respiratory viral infection-related anosmia is caused by nasal mucosa inflammation and edema, resulting in restriction of odorants' delivery to the olfactory receptors, and is associated with nasal symptoms such as congestion [36]. On the other hand, COVID-19-related anosmia usually occurs without significant nasal symptoms, and some studies showed that olfactory cleft edema may be present even in the absence of nasal congestion [37,38]. According to another hypothesis, SARS-CoV-2 does not directly affect olfactory receptor neurons (ORNs) but instead appears to target non-neuronal cells, specifically sustentacular and basal cells that express angiotensin-converting enzyme 2 (ACE2) receptors [39,40]. This leads to local inflammation and damage to the olfactory epithelium. Despite the absence of direct viral invasion of olfactory sensory neurons, their function and ability for restoration appear to be indirectly impaired by disruption to their supporting cells. It is hypothesized that early recovery occurs following rapid recovery of the sustentacular cells, allowing complete restoration of ORNs' function. In contrast, a delay in sustentacular cells' recovery can lead to the loss of the function of the ORNs. Of course, prediction of the recovery process seems challenging, as olfactory restoration may be related to multiple factors, including the regenerative capacity of the olfactory epithelium. Furthermore, delayed recovery of olfactory function may be associated with persistent inflammation and immune-mediated impairment of olfactory stem cell regeneration, as well as potential neuroinvasion through the olfactory bulb [41,42]. Coronaviruses are known to be potentially neuroinvasive, and a high proportion of affected patients experience neurological symptoms [42]. This complex interplay between peripheral epithelial injury, immune response, and potential central nervous system involvement may explain the variability in olfactory function recovery rates after COVID-19 infection. Despite a growing body of evidence, there is still a relatively poor understanding of the underlying mechanisms of COVID-19-related OD, and further progress is crucial, as it will allow targeted therapeutic modalities.

It becomes evident that effective management of these patients with COVID-19-related smell impairment is essential. Among the current therapeutic options, olfactory training is consistently recognized as the only non-pharmacological intervention demonstrating significant efficacy for post-COVID-19-related OD [43]. A standard protocol for olfactory training, as outlined by Chen et al., involves a 12-week regimen of twice-daily exposure to the following four specific odorants: rose, eucalyptus, lemon, and cloves [44]. This method demonstrates multidimensional benefits, improving threshold, discrimination, and identification scores, which suggests that it influences both peripheral and central olfactory mechanisms. The efficacy of corticosteroids has also been evaluated; a systematic review and meta-analysis found that topical but not systemic corticosteroids were effective [45]. However, combining either intranasal or oral corticosteroids with olfactory training did not provide any additional benefit over olfactory training alone [43]. Another promising treatment is a combination of micronized palmitoylethanolamide (PEA) and luteolin, known as CoUltraPEALut, which demonstrated significant efficacy in restoring olfactory function compared with conventional therapy and is proposed as a future adjuvant treatment [46]. More evidence is required to support other suggested modalities, including oral vitamin-mineral supplementation, zinc, and vitamin A, as well as platelet-rich plasma (PRP) injection into the nasal cleft [43].

This review has certain limitations, and any conclusions should be made with caution. First, a significant limitation prevalent across numerous studies arises from methodological heterogeneity, which complicates direct comparisons and comprehensive meta-analysis. A notable constraint in several studies is the frequent dependence on subjective assessment methods, potentially leading to measurement bias. Many investigations lacked an objective baseline assessment of olfactory function for all participants, thereby complicating the precise monitoring of recovery from a documented starting point; for example, objective tests, such as UPSIT, were sometimes only administered to subgroups or at subsequent follow-up intervals. A primary concern is the inherent difficulty of establishing participants' baseline olfactory function prior to COVID-19 infection, which makes it difficult to definitively distinguish between new-onset dysfunction and pre-existing olfactory disorders. Furthermore, limitations related to sample size and follow-up protocols are apparent, thus restricting the generalizability and statistical robustness of the findings. Furthermore, most studies included were of low quality. Overall, these factors underscore the necessity for more standardized and rigorous research methodology with a larger number of patients and the use of validated measurement tools to enhance the understanding of post-COVID-19 olfactory dysfunction.

## Conclusions

In conclusion, this systematic review highlights the considerable and persistent burden of OD as a sequela of COVID-19. While a high prevalence of OD is observed during the acute phase of infection, long-term studies consistently demonstrate that a significant subset of individuals continues to experience enduring or incomplete recovery over extended periods of months or even years. This quantitative loss, including anosmia and hyposmia, is frequently accompanied or succeeded by qualitative distortions, such as parosmia and phantosmia, which can emerge following a period of perceived recovery and profoundly diminish quality of life. The course of recovery is influenced by a range of factors, including the patient's age, initial severity and duration of the dysfunction, the presence of co-existing symptoms, and possibly sex.

A notable challenge within the current literature is the considerable heterogeneity in study design, assessment tools, and follow-up duration, which impede direct comparisons. Many studies rely solely on subjective patient self-reports, which often misrepresent actual recovery when contrasted with more reliable psychophysical testing. Progressing forward requires a deeper understanding of the underlying pathophysiology, which is essential for developing more targeted and effective therapeutic modalities. Future research should focus on identifying prognostic biomarkers, stratifying patient subgroups by clinical characteristics, and developing personalized follow-up strategies to facilitate timely identification, accurate prognosis, and effective management of affected individuals. Addressing current methodological, follow-up, and clinical standardization gaps will be vital not only for improving outcomes for individuals with long-term OD but also for preparing for future pandemics with similar sensory sequelae.
